# Interpersonal Physiological Synchrony for Detecting Moments of Connection in Persons With Dementia: A Pilot Study

**DOI:** 10.3389/fpsyg.2021.749710

**Published:** 2021-12-13

**Authors:** Dannie Fu, Natalia Incio-Serra, Rossio Motta-Ochoa, Stefanie Blain-Moraes

**Affiliations:** ^1^Biosignal Interaction and Personhood Technology (BIAPT) Lab, McGill University, Montreal, QC, Canada; ^2^Department of Biomedical Engineering, McGill University, Faculty of Medicine, Montreal, QC, Canada; ^3^School of Physical and Occupational Therapy, Faculty of Medicine and Health Sciences, Montreal, QC, Canada

**Keywords:** electrodermal activity (EDA), symbolic transfer entropy (STE), physiological coherence, movement, dementia

## Abstract

Interpersonal physiological synchrony has been successfully used to characterize social interactions and social processes during a variety of interpersonal interactions. There are a handful of measures of interpersonal physiological synchrony, but those that exist have only been validated on able-bodied adults. Here, we present a novel information-theory based measure of interpersonal physiological synchrony—normalized Symbolic Transfer Entropy (NSTE)—and compare its performance with a popular physiological synchrony measure—physiological concordance and single session index (SSI). Using wearable sensors, we measured the electrodermal activity (EDA) of five individuals with dementia and six able-bodied individuals as they participated in a movement activity that aimed to foster connection in persons with dementia. We calculated time-resolved NSTE and SSI measures for case studies of three dyads and compared them against moments of observed interpersonal connection in video recordings of the activity. Our findings suggest that NSTE-based measures of interpersonal physiological synchrony may provide additional advantages over SSI, including resolving moments of ambiguous SSI and providing information about the direction of information flow between participants. This study also investigated the feasibility of using interpersonal synchrony to gain insight into moments of connection experienced by individuals with dementia and further encourages exploration of these measures in other populations with reduced communicative abilities.

## 1. Introduction

Interpersonal physiological synchrony is a measure that has been used to investigate the relationship between individual's physiological dynamics and the interpersonal contexts and interactions in which they manifest. It has become a principal focus in social psychophysiology as an objective measure of the strength of interpersonal interactions across various social contexts (Palumbo et al., 2017; Helm et al., 2018). Physiological synchrony has been successfully used to characterize the relational dynamics of romantic couples (McAssey et al., 2013; Chatel-Goldman et al., 2014; Karvonen et al., 2016; Liu et al., 2016; Chaspari et al., 2017a,b; Timmons et al., 2017; Corner et al., 2019); friends and strangers (Slovák et al., 2014; Danyluck and Page-Gould, 2019; Bizzego et al., 2020); therapist-patient dyads (Marci and Orr, 2006; Marci et al., 2007; Tschacher and Meier, 2020); students working in groups (Guastello et al., 2006; Kelava et al., 2015; Mønster et al., 2016; Ahonen et al., 2018; Haataja et al., 2018; Malmberg et al., 2019); and parent-child dyads (Baker et al., 2015; Bernard et al., 2017; Bell, 2020; Samadani et al., 2021). The patterns of physiological linkages across different social contexts provide insight into social factors such as emotion, empathy, influence, conflict, and cooperation, that may not be accessible through self-reports or behavioral observations alone (Blain and McKeever, 2011; Thorson et al., 2018). As such, it has particular promise for providing insight into the interpersonal interaction dynamics of individuals who are unable to provide self-report and those who are otherwise minimally communicative (Monin and Schulz, 2009; Gifford et al., 2021).

Broadly, two main approaches have been used to quantify interpersonal physiological synchrony. The first examines the relationship between a dyad's physiological signals at the same point in time (Henning et al., 2001; Chatel-Goldman et al., 2014; Thorson et al., 2018). Cross-wavelet analysis and wavelet coherence are measures that have been adopted by several studies to detect co-synchrony of signals in the time-frequency domain (Fujiwara and Daibo, 2016; Ward et al., 2018; Gupta et al., 2019; Fujiwara et al., 2020; Hale et al., 2020). Cross-wavelet analysis, which shows the frequencies with high common power, has been used to analyze movement during dyadic conversations to investigate movement coordination between dancers and non-dancers, and to measure non-verbal social coordination in a group consisting of actors and children with autism during a theatrical workshop (Washburn et al., 2014; Fujiwara and Daibo, 2016; Ward et al., 2018; Gupta et al., 2019). Wavelet coherence highlights common frequencies regardless of power and has been used to capture synchrony in eye blinks and head nodding during face-to-face conversations. It has been used in several studies investigating neural synchronization in EEG recordings (Sun et al., 2003; Gupta et al., 2019; Nguyen et al., 2021). Physiological concordance (PC) and Single Session Index (SSI) are time-domain examples of this approach that are particularly common for measuring synchrony in electrodermal activity (Marci et al., 2007; Slovák et al., 2014; Karvonen et al., 2016; Karvonen, 2017; Di Lascio et al., 2018; Haataja et al., 2018; Gashi et al., 2019; Malmberg et al., 2019; Dindar et al., 2020; Misal et al., 2020; Liu et al., 2021). SSI is a Pearson correlation-based measure which provides an overall measure of synchrony for a particular interaction context. Several studies have adopted a rolling SSI method of analyzing moment-to-moment overall correlation of the physiological signals (Slovák et al., 2014; Haataja et al., 2018; Misal et al., 2020). The second approach uses time-lagged techniques, such as coupled differential equation models, lagged cross-correlations, and Granger causality models, to study the predictive relationship between a dyad's physiological signals (Schreiber, 2000; Müller and Lindenberger, 2011; Ferrer and Helm, 2013; Marzbanrad et al., 2015; Helm et al., 2018). Dynamical systems and coupled oscillator approaches to modeling synchrony have also been widely used to examine interpersonal synchrony (Helm et al., 2012; Ferrer and Helm, 2013; Chaspari et al., 2017a; Zee and Bolger, 2020). For example, Ferrer and Helm (2013) modeled dyadic interrelations using a system of linear equations, with parameters reflecting self-regulation and co-regulation (Ferrer and Helm, 2013; Chaspari et al., 2017a) used a coupled linear oscillator to model acoustic and physiological arousal of young couples engaging in a conflict discussion (Chaspari et al., 2017a; Zee and Bolger, 2020) also used coupled damped 183 oscillator models to explore physiological co-regulation in romantic couples (Zee and Bolger, 2020). Non-linear approaches to analyzing dynamical systems have also been explored in the context of interpersonal synchrony. Specifically, Recurrent Quantification Analysis (RQA), and its extensions such as Cross-RQA and Multidimensional-RQA, are techniques that have been used to investigate EDA synchrony in collaborating students and team collaboration using heart rate variability signals (Dindar et al., 2019; Veerabhadrappa et al., 2021). To date, few studies have examined directed, or causal influence of electrodermal activity signals in dyadic interactions (Helm et al., 2018). This approach has the potential to reveal more information about the dyadic interaction, including direction of influence, strength of influence, and time lags. These measures can be divided into two subcategories: model-based approaches and information-theoretic approaches (Lee et al., 2015). Granger Causality (GC) is a popular model-based approach that is commonly used to assess causality in time series (Müller et al., 2016). Methods like Granger Causality offer information about the coupling strength and direction; however, GC is limited due to its linear interpretation of the data (Lee et al., 2015; Müller et al., 2016). Information-theoretic approaches include transfer entropy, a non-linear extension of GC that addresses many of its limitations (Lee et al., 2015).

While interpersonal synchrony measures can be calculated across a wide range of physiological signals, those collected from unobtrusive wearable sensors offer the advantage of ecologically valid interactional data (Thorson et al., 2018). Signals of the autonomic nervous system (ANS) respond to an individual's mental and emotional state and have commonly been used to measure participant's level of arousal to study dyadic interactions (Guastello et al., 2006; Chaspari et al., 2017a,b; Haataja et al., 2018; Malmberg et al., 2019). In particular, electrodermal activity (EDA) can be unobtrusively collected from the wrist or fingertip and offers insight into the sympathetic responses of an individual. Fluctuations in the EDA signal, called electrodermal reactions (EDRs), reflect responses to various arousal stimuli (e.g., responses to emotional or affective processes, attention orienting stimuli), and are important features in affective computing technologies (Picard et al., 2001; Blain-Moraes et al., 2008).

The primary objective of this study was to apply an information theory-based measure—normalized symbolic transfer entropy (NSTE)—to interpersonal physiological synchrony using electrodermal activity signals. We compare time-resolved measures of NSTE with a traditional measure of interpersonal synchrony (SSI) in detecting observed moments of interpersonal connection. The secondary objective of this study was to assess the feasibility of using interpersonal synchrony measures for detecting moments of interpersonal connection in individuals with dementia. We hypothesized that NSTE would provide insight into interactional elements between individuals with dementia involved in a real-world, free-flow movement activity.

## 2. Methods

### 2.1. Participants

Eleven individuals participated in the movement session: five persons with dementia (age = 84.6 ± 7.49 years; three female and two male) and six able-bodied individuals (two dancers, two researchers, two care staff). The current study focuses on three case dyads (*n* = 6) including three persons with dementia (age = 89.67 ± 2.31 years; one female and two male) who were paired with three able-bodied individuals (two dancers and one researcher) during the activities. The persons with dementia had mild to moderate dementia (Alzheimer's or other dementias), but none demonstrated difficulties in comprehending the instructions and they all were aware of the time and place. All participants are identified through pseudonyms. This study was approved by the Institutional Review Board of McGill University (study number A06-B25-17B). Written consent was obtained from all caregivers and verbal assent was sought from the persons with dementia prior to and throughout the course of the study. Participants also consented to the use of photos from the movement activity for disseminating research results.

### 2.2. Description of Movement Session

**Figure 1:**
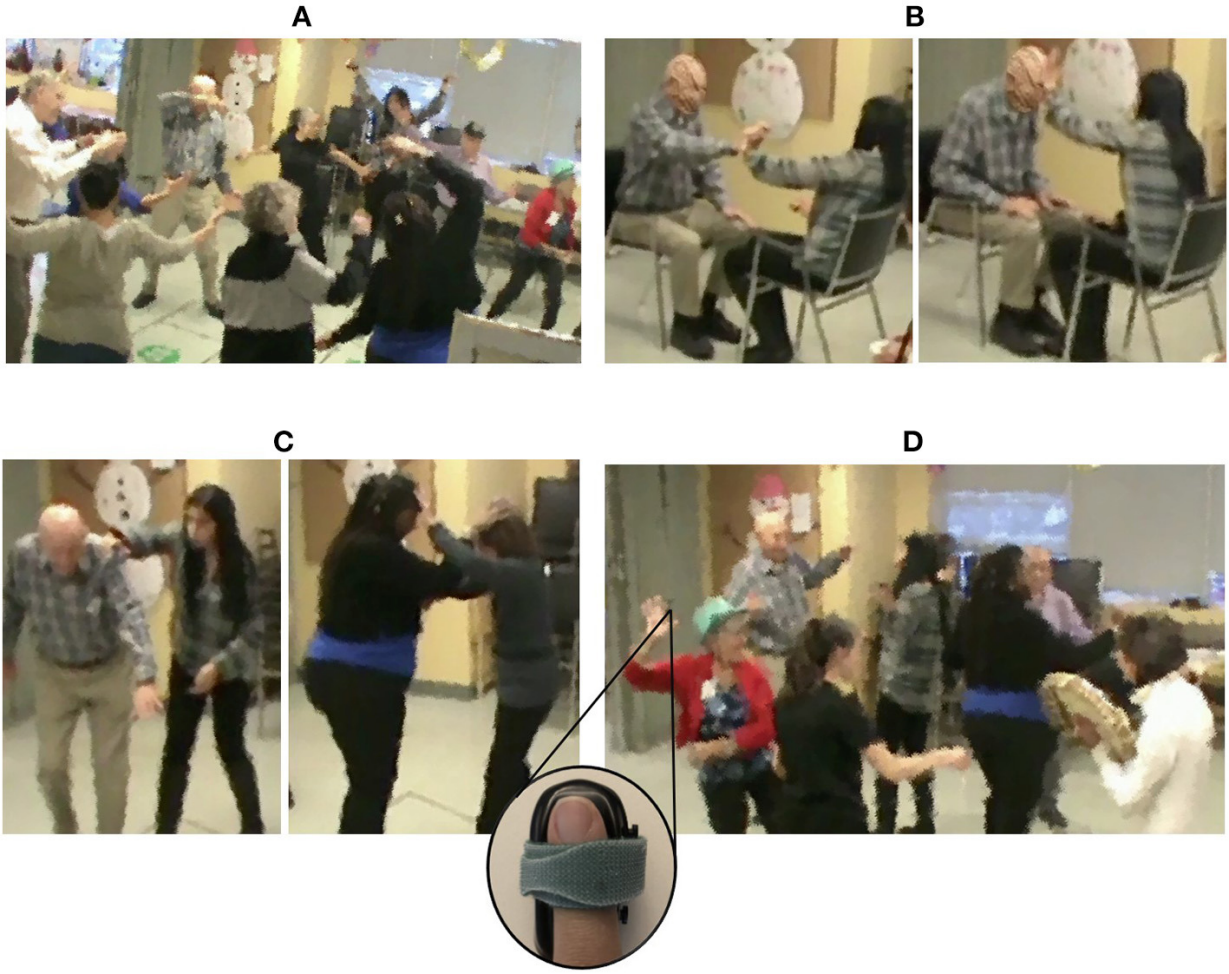
Different activities comprising the movement session: **(A)** Solo activity: musical textures. **(B)** Duo activity: Resisting the force (sitting). **(C)** Duo activity: Resisting the force (standing). **(D)** Solo activity: Improvisation with music.

The movement session took place at a non-profit organization for persons with dementia and was led by two dancers and a musician. The session lasted for 34 min and explored the theme of resistance through various activities in two solo/group and two duo exercises (Table 1, Figure 1). Participants were paired up in the same dyads for both duo activities. The lead dancer explained each of the exercises and demonstrated the movements with her own body and with the help of the supporting dancer for the duo exercises. Each exercise was accompanied by live music played by the musician using three instruments (kalimba, carillon, Indigenous drum) to structure the pace of the movements. For further description of the session, the reader is referred to (Motta-Ochoa et al., 2021).

**Table 1:** Description of activities within the session (Motta-Ochoa et al., 2021).

Musical Textures	Solo (everybody in a circle)	The musician plays three different instruments. Each instrument is associated with a movement.
Resisting the force (sitting)	Duo	Participants work in duos. They sit in chairs facing each other. One pushes different body parts of his/her partner and the partner resists with equal force.
Resisting the force (standing)	Duo	Dyads stand up and do the same exercise as the previous, moving in space, into and out of the middle of the room.
Improvisation with music	Solo (everybody in a circle)	Participants work in solos, exploring their muscular resistance when the movement flows and when it stops.

### 2.3. Data Collection

The entire movement session was video recorded through a camera mounted near the ceiling of the room. The camera used was the Sony DCR-SX63 Handycam Camcorder, which has 410k effective pixels of resolution. Video data was synchronized with physiological data collected from a battery-operated wearable sensor (Triple-Physiology Sensor (TPS), Thought Technology Ltd.,) that all participants wore on their fingertip or palm. The sensor is powered by a Lithium-ion polymer battery with a nominal voltage of 3.7V. The physiological sensor captured three autonomic nervous system (ANS) signals: (1) electrodermal activity (EDA), (2) skin temperature, (3) blood volume pulse (BVP). These signals were measured by the TPS at 15, 15, and 300 Hz, respectively, and were transmitted wirelessly using Bluetooth to an Android phone. Changes in these physiological signals are connected to emotional or mental states, and reflect not only changes due to body movement, but also mental and emotional state (Blain-Moraes et al., 2008; Collet et al., 2013). We focused our analysis on EDA, as this signal has been used extensively in previous research to measure physiological synchrony (Palumbo et al., 2017). EDA measurement from the TPS ranged from 0 to 30 μS.

### 2.4. Data Analysis

#### 2.4.1. Qualitative Video Analysis

Two ethnographers who participated in the movement session comparatively analyzed fieldnotes of participant observations and video recordings to identify moments of interpersonal connection. Videos were reviewed multiple times in an iterative fashion to identify how each participant established connections. Moments of interpersonal connection involved a mutual exchange between two or more participants and were required to meet the following criteria: (1) sustained for 30 s or longer; (2) had a clear beginning and end; and (3) involved the participants focusing his/her attention on another participant or the group. To ensure validity, four researchers not involved in the movement program analyzed more than 10% of the collected material using these criteria, with an inter-rater agreement of 100%. For more details, the reader is referred to (Motta-Ochoa et al., 2021).

#### 2.4.2. Physiological Synchrony Analysis

Prior to computing physiological synchrony, the EDA signals were pre-processed to remove non-physiological artifacts. A 1-D median filter (order *n* = 75) and moving average filter with a 0.75 s window were applied to smooth the data, and a one-Euro filter was applied to the EDA signal to minimize jitter and lag (Lai Kwan et al., 2019). The smoothed EDA was then used to calculate two measures of physiological synchrony across all dyads: (1) Single Session Index (SSI) (Marci et al., 2007) and (2) normalized Symbolic Transfer Entropy (NSTE).

##### 2.4.2.1. Physiological Concordance (PC) and Single Session Index (SSI)

The EDA signals of both members of the dyad were first Z-score standardized to make them more comparable (Ben-Shakhar, 1985; Haataja et al., 2018). The average slopes of the EDA signal were computed over a 5-s sliding window with a step-size of 1 s. Physiological concordance (PC) was determined by computing consecutive Pearson correlations between the two slopes using a 15-s sliding window with a step-size of 1 s (Marci and Orr, 2006; Marci et al., 2007). The single session index (SSI) was calculated by taking the natural log transform of the ratio of the sum of the positive correlations over the sum of the negative correlations:


(1) $$\begin{array}{c} SSI=ln\frac{\sum_{R>0}R}{\sum_{R<0}\left|R\right|} \end{array}$$

We computed the SSI over a 60-s sliding window to analyze the time-resolved interpersonal synchrony across the different activities of the movement session (Slovák et al., 2014). Significance of the moment-by-moment SSI was assessed through Monte Carlo shuffling, which has been similarly applied in previous studies on the PC index (Karvonen et al., 2016). Within a given dyad, the Pearson correlation was computed on the two EDA signals where the time series of one participant was preserved, while that of the second participant was shuffled. This procedure was repeated 1,000 times. Observed SSI values were compared to the distribution of shuffled SSI values, and were considered statistically significant if they exceeded the top 5%.

##### 2.4.2.2. Normalized Symbolic Transfer Entropy (NSTE)

Transfer Entropy (TE) is a non-linear extension of Granger Causality (GC). It is a model-free, information theory-based approach to estimating both the strength and direction of connectivity between a target signal (Y) and source signal (X) (Lee et al., 2013, 2015). Transfer Entropy is defined as the amount of mutual information between the past of X (X^P^) and the future of Y (Y^F^), when the past of the Y is known (Lee et al., 2015):


(2) $$\begin{array}{l} TE_{X\to Y}=I\left(Y^{F};X^{P}|Y^{P}\right)=H\left(Y^{F};Y^{P}\right)-H\left(Y^{F};X^{P},Y^{P}\right), \end{array}$$

where *H*(*Y*^*F*^|*Y*^*P*^) is the entropy of the future of Y given its past. That is, if adding the past of the source signal allows us to better predict the future of the target signal, the source has a causal relationship with the target. Symbolic transfer entropy (STE) improves upon TE through symbolizing the vectors for the past of X, the past of Y, and the future if Y. Three parameters define the computation of STE: (1) the embedding dimension (*d*_*E*_) specifies the length of the vector; (2) the time delay (τ) which specifies the time between vector points; and (3) the prediction time (δ), which defines the delay between the source and target signal (Staniek and Lehnertz, 2008; Lee et al., 2015).

To account for potential spurious STE values, we shuffled the source signal (X) while leaving the target signal (Y) intact, such that the frequency distribution and signal characteristics remained the same, but the dependency of *X*^*P*^ and *Y*^(*F*|*P*)^ was removed (Gouritch and Eggermont, 2007; Lee et al., 2013, 2015). We repeated this procedure 20 times to produce $STE_{X\to Y}^{shuffled}$—an estimation of the bias produced by the signal characteristics of the source signal (X). The STE was then normalized to account for the bias incurred if the target signal (Y) exhibits high autocorrelation (Lee et al., 2015):


(3) $$\begin{array}{l} NSTE_{X\to Y}=\frac{STE_{X\to Y}-STE_{X\to Y}^{shuffled}}{H\left(Y^{F}|Y^{P}\right)}\in \left[0,1\right]. \end{array}$$

Finally, we assessed the direction of information flow between members of the dyad by computing the asymmetry of the NSTE originating from each participant:


(4) $$\begin{array}{l} Asymmetry_{X\to Y}=\frac{NSTE_{X\to Y}-NSTE_{Y\to X}}{NSTE_{X\to Y}+NSTE_{Y\to X}}\in \left[-1,1\right]. \end{array}$$

When *asymmetry*_*X*→*Y*_ is positive, information predominately flows from person X to person Y, and vice versa when the value is negative. In this study, person X was always set as the able-bodied adult and person Y was always the individual with dementia.

*NSTE*_*X*→*Y*_ was computed over 60 s sliding windows, and the average asymmetry was computed over 5 sliding windows from the NSTE signal. For the computation of STE, we systematically searched a broad parameter space for the set of parameter values that produced the maximum NSTE for each dyad. The embedding dimension (*d*_*E*_) was fixed at 3, the time delay (τ) was chosen to produce the maximum NSTE from a range of τ=15 to τ=120 (1 to 8 s), and the prediction time (δ) was determined with the time lag resulting in the maximum cross-correlation from 1 to 50 (0.07–3.33 s).

**Figure 2:**
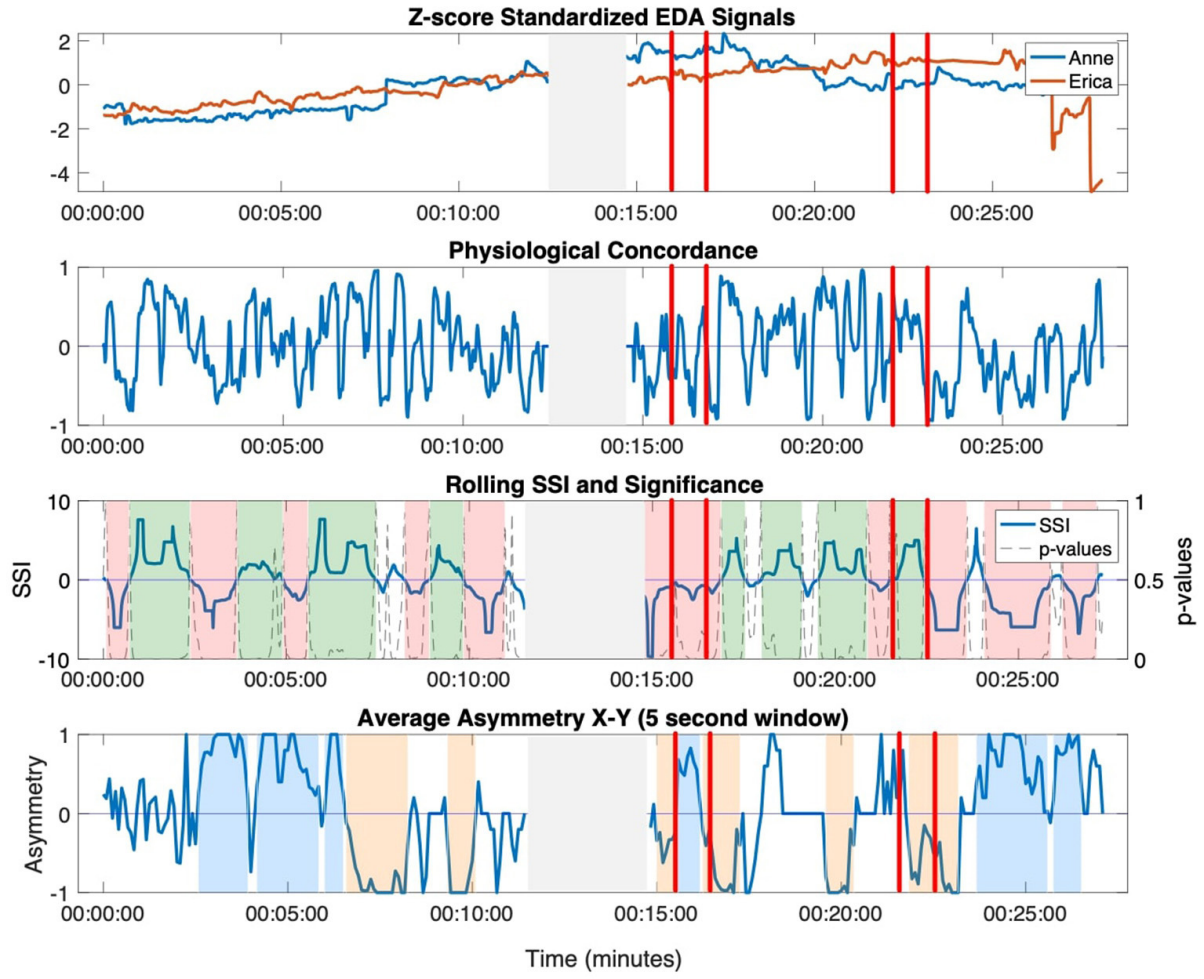
Standardized EDA signals, physiological concordance, SSI, and asymmetry for an example dyad across the entire movement session. Physiological concordance was computed over a 15 s moving window; the SSI was computed over a 30 s moving window; and the *asymmetry*_*X*→*Y*_ was computed as the average NSTE (computed with a 60 s moving window) in a 5 s window. Moments of interpersonal connection identified by the ethnographers are shown between the vertical red lines. Moments of sustained synchrony (at least 30 s) are highlighted in green (sustained positive SSI), red (sustained negative SSI), blue (sustained *asymmetry*_*X*→*Y*_), orange (sustained *asymmetry*_*Y*→*X*_). The gray shaded area in the middle of the session indicates the time interval where the sensor disconnected.

Standardized EDA and the two time-resolved measures of interpersonal synchrony across the full movement session are presented in Figure 2 for an example dyad.

#### 2.4.3. Data Triangulation: Physiological and Video Analysis

As we have previously validated the observed moments of interpersonal connection identified in the video analysis (Motta-Ochoa et al., 2021), our primary data triangulation used results from the video observation to frame the physiological analysis. Specifically, we segmented the SSI and NSTE time series according to the moments of connection identified in video analysis, for the three case dyads. This ensured that we extracted all measures of physiological synchrony that corresponded to observed interpersonal connection. Within each of these segmented windows, we compared the dynamic patterns of SSI and NSTE to assess the characteristics of each metric during moments of interpersonal connection. As a secondary triangulation, we used the physiological results to frame the video analysis. Sustained moments of high SSI and high NSTE were time-stamped in the video; movements and interactions of the corresponding dyad were re-examined for potential sources of the high physiological synchrony.

## 3. Results

**Figure 3:**
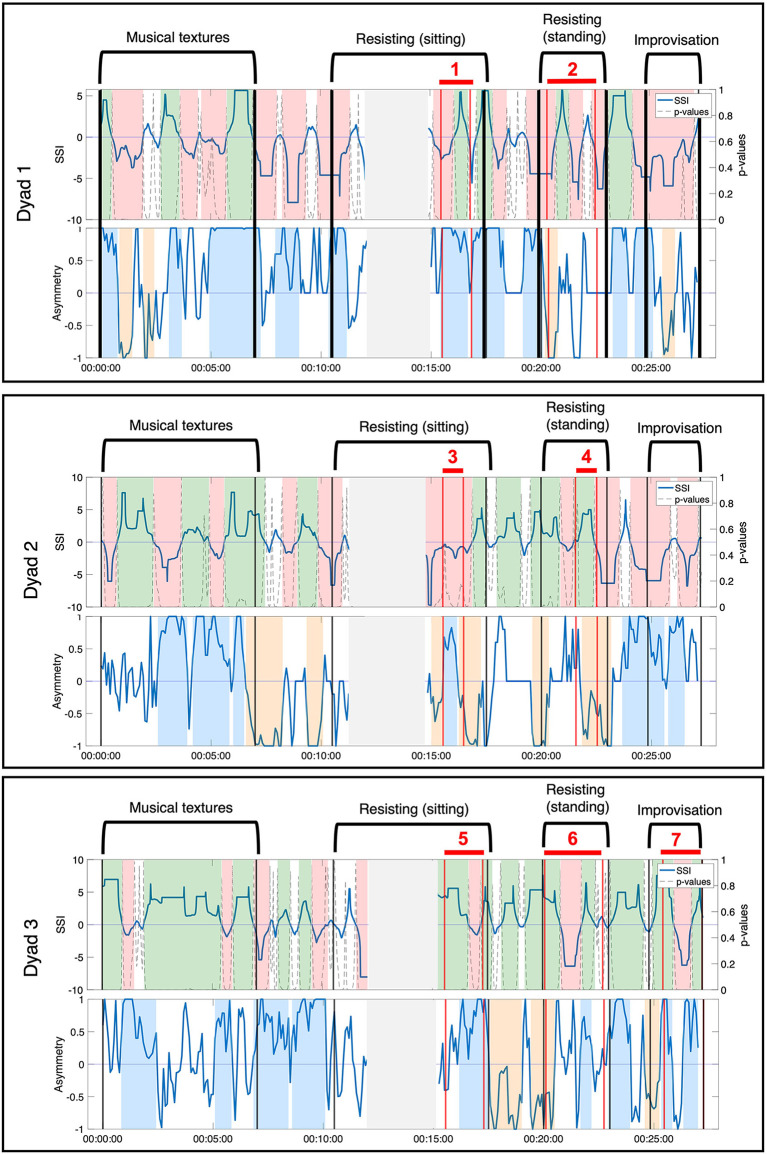
Annotated movement sessions for the three dyads (Dyad 1: Hector and Jean, Dyad 2: Erica and Anne, Dyad 3: Ruth and Harvey). Observed moments of connection are labeled and shown between the vertical red lines. Moments where the sensor disconnected are shown in the gray shaded area.

Through the video analysis, a total of seven moments of interpersonal connection were identified across three dyads. Detailed descriptions of the moments of connection are presented in Table 2. Moments of interpersonal connection observed in the video predominantly occurred during the duo activities, with one occurring during the improvisation with music activity. While this improvisation was designed as a solo exercise, the video showed that members of the group spontaneously split into pairs and danced, effectively rendering this last moment a duo activity as well. Moment 1 and Moment 2 occurred during the resistance sitting and resistance standing activity between Jean (female, dancer) and Hector (male, person with dementia). Moment 3 and Moment 4 occurred during the resistance sitting and resistance standing activity between Anne (female, dancer) and Erica (female, person with dementia). Moment 5, Moment 6, and Moment 7 occurred during the resistance sitting, resistance standing, and improvisation activity, between Ruth (female, researcher) and Harvey (male, person with dementia). The two physiological synchrony metrics—SSI and NSTE—were calculated across the entire movement session for each dyad that had observed moments of interpersonal connections. The time-resolved physiological synchrony annotated with the movement session activities and moments of interpersonal connection are presented in Figure 3.

**Table 2:** Description of moments of connection from video analysis.

	**Dyad**	**Activity**	**Time stamp**	**Description**
1	Hector – Jean	Resisting the force (sitting)	15.30"–16.50"	The dyad is engaging in mutual resistance; they are connected at the wrists, pulling and pushing like waves. Jean follows the movement of their wrists with her eyes, head, body; she is dynamic in her movements - her wrists twist around Hector's, and her body raises and lowers with the flow.
2	Hector – Jean	Resisting the force (standing)	20.20"–22.32"	Jean has her hand on Hector's hand, resisting as he moves into the middle; she transitions her hand placement to his shoulder, and he slowly pushes against her hand out of the middle. They switch roles and Jean beckons Hector to place his hand on her shoulder while she moves into the middle of the circle. At the middle of the circle, she gestures to him to place his hand on another part of her body again to resist against her as she moves out of the middle. She is dynamic with her movements as she twists her body around Hector's hand as she moves into and out of the middle. They switch roles again; Jean places her hand on the side of Hector's head as he pushes against her hand into the middle. In the middle, Jean switches to place her hand on his hand and resists against him as he walks out of the middle. They switch for the final time, with Jean moving in and out of the middle.
3	Erica – Anne	Resisting the force (sitting)	15.32"–16.28"	They are both engaged in mutual resistance; similarly to Jean and Hector, their wrists are connected as they move their arms up and down, left and right. They both move their bodies with the movement of their arms; as they bring their arms down, their bodies move down, as they bring their arms left and right, their bodies do the same. Erica is quiet as she concentrates on the movements; they find a fluidity together.
4	Erica – Anne	Resisting the force (standing)	21.34"–22.32"	Anne has her hand placed on different locations on Erica's body as Erica pushes against her around the room. First, Anne has her hand on Ericas hip while Erica pushes against her into the middle. Then, they connect at the hand and Anne resists as Erica pushes against her out of the middle. Anne places her hand on Erica's back and Erica slowly walks backwards, pushing against her hand. Finally, Anne resists Erica's pushing into the middle by placing her hand on Erica's forehead. Erica seems very attentive; she focuses on the activity and does not laugh.
5	Ruth – Harvey	Resisting the force (sitting)	15.33"-16.17"	Their hands are clasped together, pushing back and forth, sometimes both hands pushing and pulling, other times alternating which hand is forwards and back. In the beginning, it appears as if Harvey is pushing strongly against Ruth, as her back is pressed against the chair while his body is leaning forwards. As the activity progresses, Ruth pushes forward and they meet in the middle; their hands move back and forth, and they occasionally sway left and right.
6	Ruth – Harvey	Resisting the force (standing)	20.06"-22.44"	Ruth is pushing against Harvey's shoulder. She repeats the instructions several times during the exercise. From time to time, she tells Harvey that he is very strong. It does not appear that Ruth is resisting very hard against Harvey; she appears to be guiding him. They switch roles, and Harvey places his hand on Ruth's shoulder as she moves into and out of the middle. They switch roles again; Ruth places her hand on Harvey's forehead as he bends forward and pushes against her hand into and out of the middle. At the end, Ruth holds one of Harvey's hands with her two hands and tells him: Very good!.
7	Ruth – Harvey	Improvisation with music	25.28"-27.15"	Ruth and Harvey are dancing in front of each other to the beat of the drums, slowing down when the drums slow, speeding up when the drums speed up, and freezing when the drums stop. Harvey starts mirroring Ruth's movements; as she raises her arms, he raises her arms, as she brings her arms in, as does he. When Ruth leans to her left, he leans to his right.

### 3.1. NSTE Asymmetry Provides Complementary and Additional Information to SSI About Moments of Interpersonal Connection

Patterns of NSTE asymmetry and SSI were compared during each observed moment of interpersonal connection to assess the characteristics of the two measures of physiological synchrony. In comparison to the established metric of SSI, NSTE asymmetry provided complementary and additional information that informed the interpretation of each of these moments of interpersonal connection. We illustrate the relationship between the two metrics by describing four particular ways that NSTE asymmetry provides additional insight to SSI during a moment of connection.

#### 3.1.1. Case 1: During Sustained Positive SSI, NSTE Asymmetry Informs Direction of Information Flow Within a Dyad

**Figure 4:**
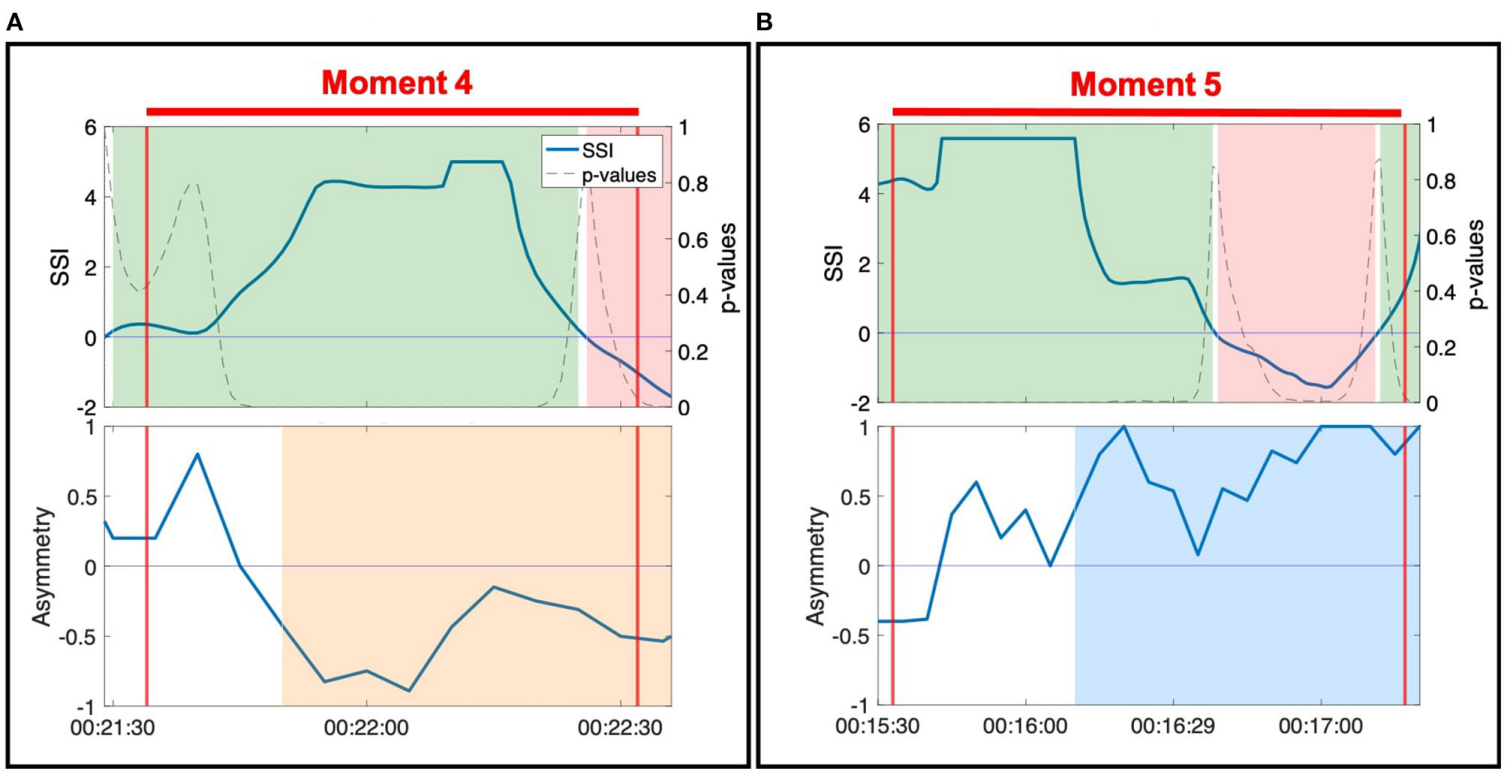
SSI and NSTE asymmetry values during two moments of connection: **(A)** Moment 4: Anne and Erica during the resistance standing activity. **(B)** Moment 5: Ruth and Harvey during the resistance sitting activity.

During Moment 4 and Moment 5, the SSI of the dyad's EDA was sustained at a statistically significant positive value for the majority of the interaction (Figure 4). In this case, SSI indicates high interpersonal physiological synchrony in agreement with the observed interaction of the dyad in the video (Slovák et al., 2014). NSTE asymmetry values provide additional information about these moments of synchrony. In Moment 4, the NSTE asymmetry was negative, indicating that the direction of information flow was from Erica to Anne. This corresponded well to the dyad's interaction in the video, where Erica led the interaction of pushing against Anne's hand so that the dyad moved into and out of the middle of the room. In Moment 5, the NSTE asymmetry was positive, indicating that the direction of information flow went from Ruth to Harvey. In reviewing the video recording of this moment, both members of the dyad were attending to each other and appeared engaged in the activity. While the direction of information flow cannot be confirmed through behavioral observation, it is consistent with the typical interactions within this dyad, where Harvey (an individual with dementia) typically follows Ruth (a researcher and also a volunteer at the organization).

#### 3.1.2. During Fluctuating, Significant SSI, NSTE Asymmetry Informs Leader-Follower Relationships Within a Dyad

**Figure 5:**
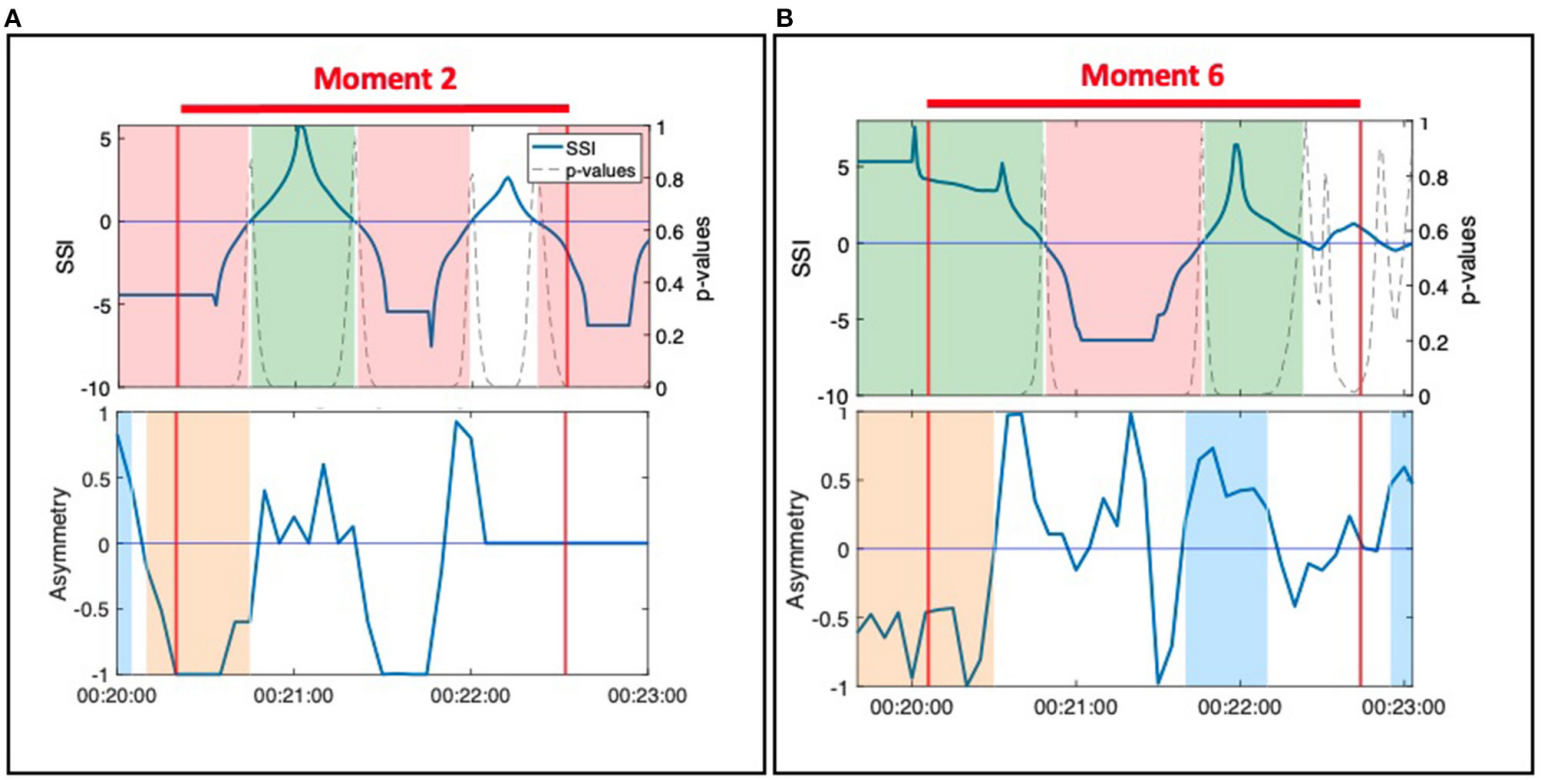
**(A)** Moment 2: Hector and Jean during the resistance standing activity. **(B)** Moment 6: Ruth and Harvey during the resistance standing activity.

During Moment 2 and Moment 6, SSI remained statistically significant throughout the observed moment of interpersonal connection but fluctuated between positive and negative values (Figure 5). Interpretation of these SSI fluctuations is informed by the NSTE asymmetry. During Moment 2, NSTE asymmetry also fluctuates, mirroring the pattern of the SSI. Here, the correlation of SSI and NSTE asymmetry reflect the shifting leader-follower roles within the dyad observed in the video. When both SSI and asymmetry were negative, Hector led Jean walking into and out of the middle; when both SSI and asymmetry were positive, Jean led Hector in this activity. In contrast, during Moment 6, SSI fluctuations are not matched by NSTE asymmetry, which remain positive for the majority of this moment of connection. Though the changes in SSI correspond with the observed activity in the video (i.e., when SSI is positive, Ruth is resisting against Harvey; when SSI is negative, Harvey is resisting against Ruth), the NSTE asymmetry indicates that information is flowing from Ruth to Harvey most of the time. This interpretation is well-supported by fieldnotes taken by the ethnographer: during this activity, Harvey was having difficulty understanding the instructions and Ruth was instructing and guiding him in performing the activity.

#### 3.1.3. Case 3: NSTE Asymmetry Identifies a Moment of Interpersonal Connection When SSI Does Not

**Figure 6:**
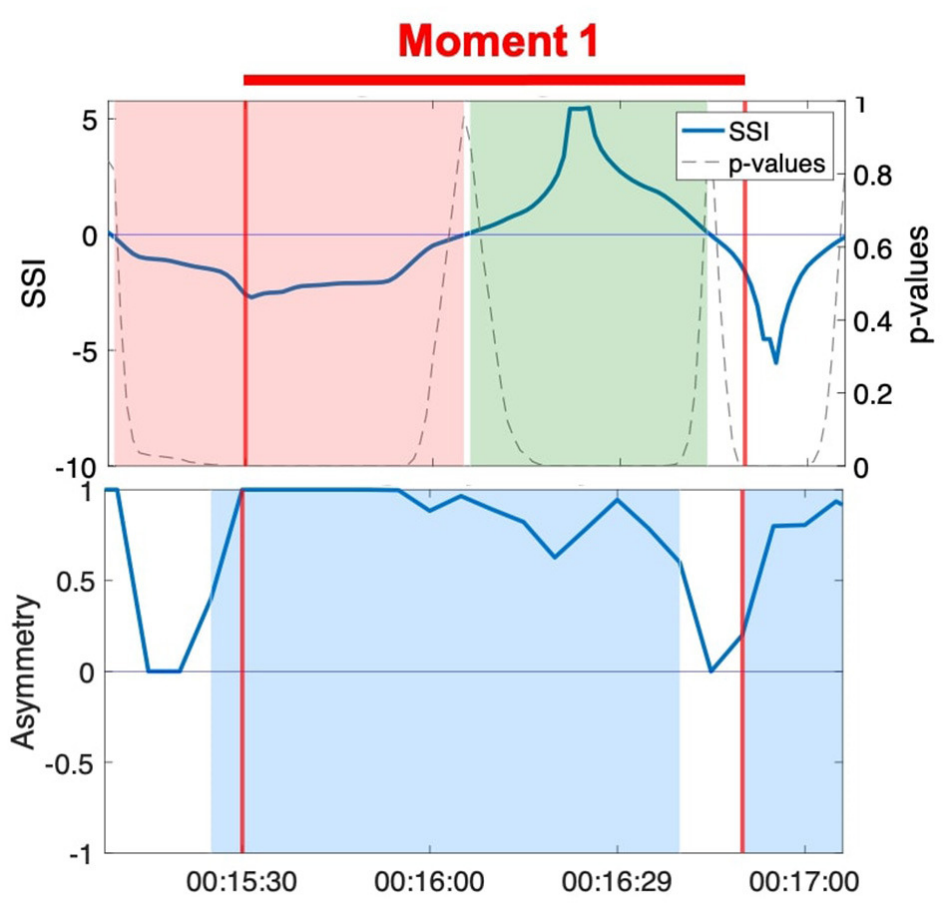
SSI and NSTE asymmetry during Moment 1: Hector and Jean during the resistance sitting activity.

The SSI during Moment 1 shifts from a significant negative value to a significant positive value halfway through the interaction (Figure 6). Unlike in case 2, the shift in SSI does not correspond to any observed or reported change in role between the members of the dyad, rendering the fluctuations in SSI unrelated to the moment of interpersonal connection. In contrast, the NSTE asymmetry remains sustained at a high level for over a minute during Moment 1. The positive NSTE asymmetry indicates information flow from Jean to Hector throughout this moment of connection, which corresponds to the observed interaction in the video recording of this interaction. In this case, the sustained high NSTE asymmetry was able to identify a moment of interpersonal connection, while the SSI was not.

#### 3.1.4. Case 4: NSTE Asymmetry Identifies a Moment of Interpersonal Connection When SSI is Negative

**Figure 7:**
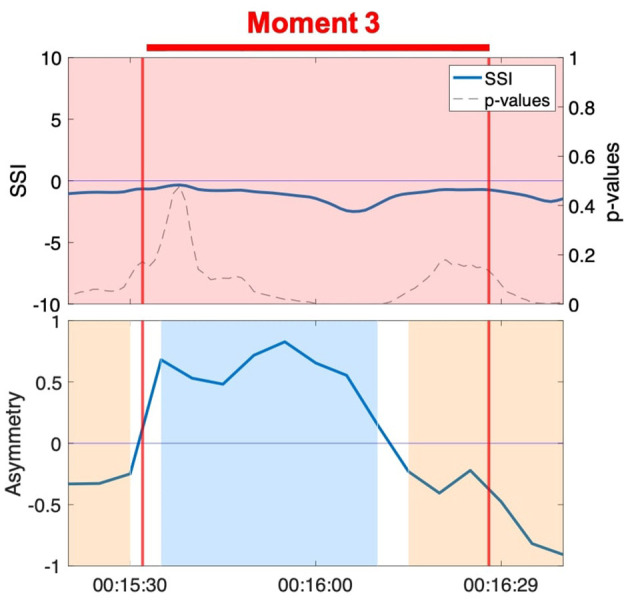
SSI and NSTE asymmetry during Moment 3: Anne and Erica during the resistance sitting activity.

Moment 3 is characterized by a sustained negative SSI value throughout the duration of the moment, though not always significant (Figure 7). Although high positive SSI has been associated with aspects of social interaction in previous research, interactions characterized by negative SSI have received little attention to date. A negative SSI indicates predominantly negative correlations between the members of the dyad (e.g., as one increases, the other decreases) (Karvonen et al., 2016). While this metric does not mark the moment of interpersonal connection, the NSTE asymmetry remains high and sustained throughout the interaction. The positive NSTE asymmetry indicates information flow from Anne to Erica, which corresponds to the interaction dynamics observed in the video. In this case, the negative SSI does not canonically reflect the moment of interpersonal connection, while the sustained high NSTE asymmetry does.

### 3.2. Spurious Sustained SSI and NSTE Asymmetry Can be Generated by a Shared Third-Party Influence

**Figure 8:**
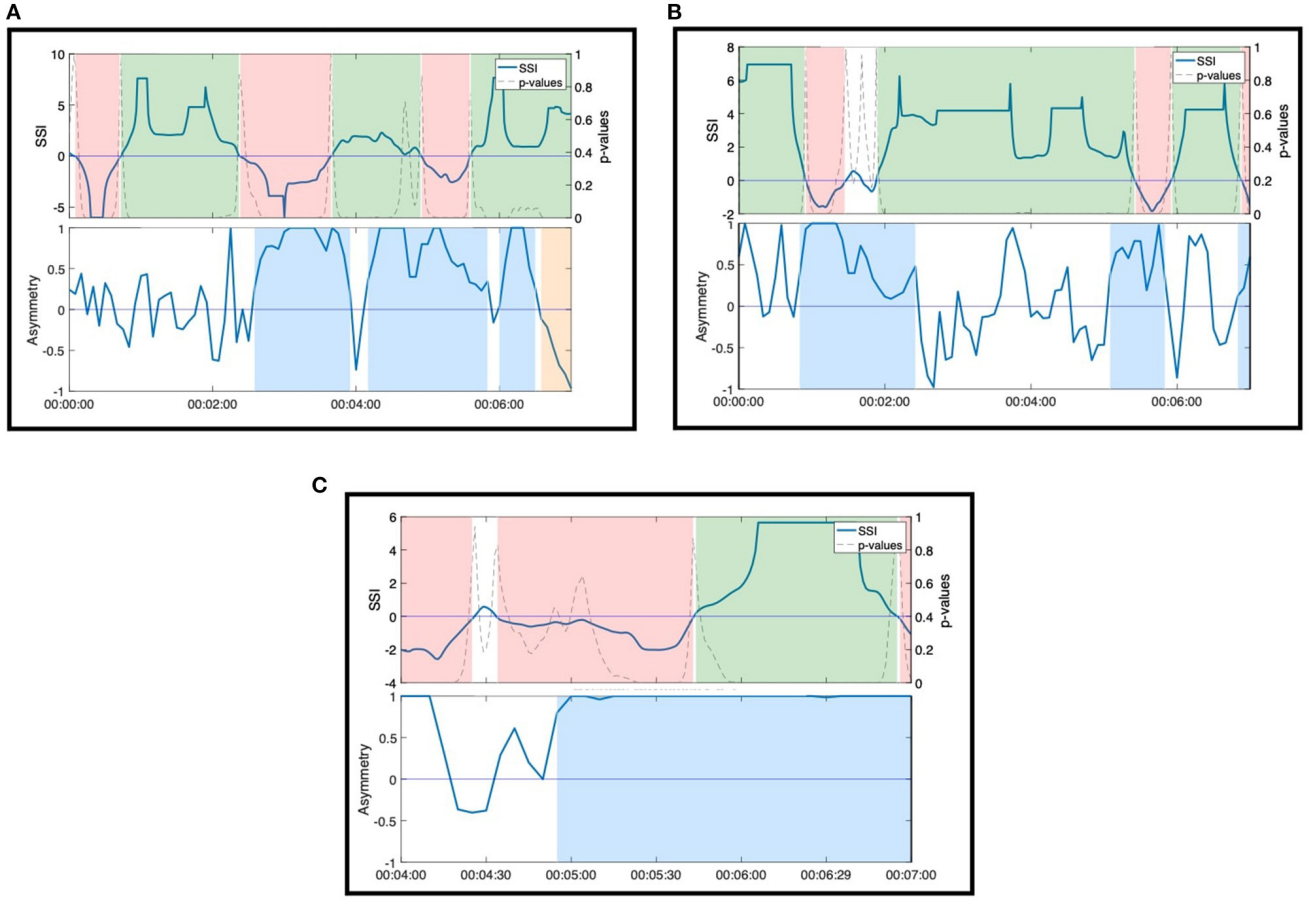
Moments of significant SSI and sustained NSTE asymmetry during non-dyadic interactions in the musical textures activity. **(A)** Anne and Erica. **(B)** Ruth and Harvey. **(C)** Jean and Hector.

In our secondary triangulation, we examined the SSI and NSTE asymmetry time series for sustained, significant moments that did not correlate to moments of interpersonal connection observed in the video. If there were no interpersonal interactions between the members of the dyad from whom the EDA was recorded at these time points, this would indicate the potential for SSI and NSTE asymmetry to generate false positive indications of physiological synchrony. We observed several instances of spurious sustained SSI and NSTE asymmetry, particularly during the musical textures activity (see Figure 8 for examples). While the musical textures activity was a solo activity, thereby precluding interpersonal moments of connection, the dancer Anne led the group in coordinated movements during these moments. SSI fluctuates inconsistently across two of the three moments (Figures 8A,C), but the NSTE asymmetry is consistently high, indicating information flow from the dancer or able-bodied person to the person with dementia. The third moment (Figure 8B) shows a consistently positive SSI and fluctuating, but predominantly high NSTE asymmetry, corresponding well to their engagement in the activity observed in the video.

## 4. Discussion

The current study presents a novel measure of interpersonal physiological synchrony—normalized symbolic transfer entropy (NSTE)—and demonstrates its potential for detecting moments of interpersonal connection via electrodermal activity. In comparing NSTE to SSI (a more established measure of interpersonal physiological synchrony) during observed moments of connection, we demonstrate that NSTE asymmetry provides complementary and additional information that informs the interpretation of the significant moments of interpersonal interaction. This is also the first study to demonstrate the feasibility of using interpersonal physiological synchrony to detect significant moments of connection in individuals with dementia in a naturalistic environment.

NSTE, transfer entropy and other symbolic analysis techniques have been reliably used in other contexts to assess information flow of biosignals within and between individuals (Lee et al., 2013, 2015; Shovon et al., 2014; Marzbanrad et al., 2015; Müller et al., 2016; Takamizawa and Kawasaki, 2019). Within an individual, symbolic dynamics have been used to assess heart rate variability (Guzzetti et al., 2005; Cesarelli et al., 2012); normalized transfer entropy to measure information transfer between auditory cortical neurons (Gouritch and Eggermont, 2007); and lag-specific transfer entropy in cardiovascular and cardiorespiratory signals to describe alterations in the cardiovascular and respiration mechanisms (Faes et al., 2014). Symbolic transfer entropy has been used in many studies investigating feedforward and feedback connectivity within an individual's brain (Ku et al., 2011; Lee et al., 2013, 2015). These studies have shown the potential of STE for monitoring brain states in surgical patients (Lee et al., 2015). Transfer entropy has been successfully used in biosignals to measure information flow between individuals. Marzbanrad et al. (2015) applied TE to maternal and fetal heart rates to quantify directed interactions between mother and fetus at various time delays and gestational ages. Takamizawa and Kawasaki (2019) used TE to identify the causal relationship between two people (leader and follower) during a cooperative alternative-tapping task. In both computer simulation and human behavioral experiments, TE was able to reliably identify leader and follower interpersonal relationships (Takamizawa and Kawasaki, 2019). The current study builds upon this body of research, extending transfer entropy-related measures to electrodermal activity—a biosignal that reflects the activity of the sympathetic nervous system and reflects an individual's mental and emotional state. We demonstrate that normalized symbolic transfer entropy calculated across the EDA of two individuals is related to significant moments of interpersonal connection within the dyad and can indicate the direction of information flow (e.g., leader-follower relationships) during these moments. In addition, as this measure captures increasing and decreasing patterns in the signal through symbolization of the signal values, rather than the absolute values themselves, we have seen the potential of its use as a translational measure of interpersonal synchrony for capturing relevant features of an individual's emotional and mental response rather than features related to the maintenance of homeostasis (Blain-Moraes et al., 2008).

We compare the performance of NSTE asymmetry to a more traditional measure of physiological synchrony: the single session index. Introduced by Marci and Orr (2006) with several more recent modifications (Slovák et al., 2014; Karvonen et al., 2016; Haataja et al., 2018; Malmberg et al., 2019; Misal et al., 2020; Stuldreher et al., 2020), the SSI has been well-established with electrodermal activity signals. Previous work has demonstrated that high SSI measured from EDA signals between individuals have been associated with high levels of dyadic and group emotional engagement. During real-world conversations between 20 pairs of friends, consistently high EDA synchrony (30 to 40 s) was associated with high dyadic emotional engagement, while inconsistent or fluctuating EDA synchrony was associated with low emotional engagement (Slovák et al., 2014). In clinical sessions, high SSI between patient and therapist EDA was positively associated with patient perception of therapist empathy. Observer ratings of 1-min audio-video segments showed more positive social-emotional responses during high skin conductance concordance (Marci et al., 2007). SSI has also been used to examine group physiological synchrony. Student groups have high physiological synchrony values when monitoring is frequent (Haataja et al., 2018), and when they faced difficulties during a collaborative exam (Malmberg et al., 2019). The strong association between SSI and emotional engagement demonstrated in these previous studies underscores the potential of NSTE, which provides complementary and additional information to the SSI in the current study. NSTE asymmetry could identify the direction of information flow between connected dyads in moments marked by SSI (case 1 and 2) and identified moments of interpersonal connection when SSI was ambiguous (case 3 and 4). It is worth noting both measures employed in this study are based on different fundamental concepts of interpersonal synchrony: correlation (SSI) and information theory (NSTE). Correlation-based methods analyzed concurrent synchrony at a single time point across time, while information theory-based methods examine the predictive power of the transmitter or receiver signals on their respective counterpart (Helm et al., 2018). Our results suggest that the combination of these two types of synchrony measures provide deeper insight into dynamics of social interactions and connection.

Previous studies using SSI to investigate interpersonal physiological synchrony have focused predominantly on moments of sustained, positive SSI. In our study, we observed several moments of sustained negative SSI, both associated and not associated with an observed moment of interpersonal connection. During moments of negative SSI (Moments 2, 3, and 6) both members of the dyad appeared to be engaged. This “negative synchrony” does not necessarily imply disengagement; it is possible that negative SSI measures are generated when one member of the dyad is extremely focused (inducing more EDRs), while the other member is more relaxed (Bailey, 2017). This hypothesis is supported by a handful of previous findings. Karvonen et al. (2016) and Karvonen (2017) reported a negative SSI in a particular case study of a couple during therapy, noting that when one person became more relaxed, the spouse became more alert. The low synchrony did not correspond to disengagement: it could be caused when one person was becoming agitated and the other was trying to stay calm (Karvonen et al., 2016; Karvonen, 2017). Misal et al. (2020) examined EDA synchrony using a rolling SSI in paramedic trainee pairs during *in-situ* training. They reported a higher percentage of the use of the word “we” during sessions associated with positive SSI and a higher percent usage of the word “you” during sessions associated with negative SSI (Misal et al., 2020), suggesting that negative SSI may correspond to less social cohesion.

Although the current study clearly demonstrates the potential of NSTE for the detection of moments of connection, several challenges need to be addressed before it can be implemented as a reliable measure of interpersonal physiological synchrony. Foremost among these challenges is the ability for NSTE to be confounded by a shared third source. We observed several examples of sustained high NSTE asymmetry indicating strong information flow from the lead dancer to the participants during group activities. Although the group was engaged in similar physical movements during these activities, they were executed solo, without an intentional connection to another group member. While it is known that “physiological influence may reflect exposure to shared stimuli or similar physiological milieu and not a social process between two people,” this is a potential confound that needs to be addressed through careful future study design and interpretation (Thorson et al., 2018).

The study also demonstrates the feasibility of using physiological signals to detect moments of interpersonal synchrony in individuals with dementia. One of the most insidious consequences of dementia is the rupture of relationships that often co-occurs with declining functional and cognitive abilities. Relationships between the individual with dementia and their carers often undergo severe strain when speech and communication change. This may in part be due to caregivers and family members intuitive dependence on language as a sign of emotional connection (Duffy, 1999). As a result, individuals with severe dementia often find themselves increasingly excluded from the social world. This fracturing of relationships at intimate and community levels contributes to the loss of personhood experienced by the individual with dementia and their carer (Kitwood, 1997). Techniques that can detect moments of interpersonal synchrony between an individual with dementia and their carer have the potential to mitigate this loss of personhood by manifesting otherwise-hidden moments of connection (Gifford et al., 2021). This study demonstrates that such a technology is feasible with this population, providing the first step toward the development of systems that use physiological synchrony to sustain the relationships and personhood of individuals with minimal communicative abilities.

This study has several limitations. First, the video recording of the session did not always capture all participants in the room; it is possible that several moments of interpersonal synchrony were missed because one of the participants was out of frame. Second, our identification of moments of interpersonal connection was based on iterative video analysis and ethnographic fieldnotes, rather than participant self-report. While some studies have shown that it is possible to detect interpersonal signals from non-verbal streams, and that macro-judgements may be more relevant and realistic for social signal processing than specific behavioral cues, this method may not have captured all subjective experiences of interpersonal connection (Ambady et al., 2000; Slovák et al., 2014). Third, this study was conducted with a small sample size of *n* = 6 individuals (three dyads). Although this was sufficient to accomplish the study objective of illustrating the potential of a new measure of interpersonal synchrony, future work needs to validate the reliability of this measure across a larger group of individuals. Fourth, we explored only a single physiological measure—EDA—as this is commonly used for measuring synchrony and emotional arousal. Individuals have different dominant physiological modalities and may manifest synchrony in other biosignals (e.g., heart rate) (Lai Kwan et al., 2019). Other studies have shown that performance of a multimodal physiological synchrony measure was more robust than a single physiological metric across different types of stimuli, even though the maximum performance did not improve over a single physiological metric (Verma and Tiwary, 2014; Stuldreher et al., 2020).

## 5. Conclusion

This study presented a new measure of interpersonal physiological synchrony—normalized symbolic transfer entropy—and demonstrated that it has the potential to provide complementary and additional information to the single session index in detecting moments of interpersonal connection. NSTE asymmetry provided insight about the direction of information flow within a dyad during moments of connection and detected some moments that SSI did not. These results suggest that information-theory based measures warrant further exploration in the field of interpersonal synchrony. They also suggest the potential of using this technique to detect interpersonal physiological synchrony between individuals with dementia and their carers.

## Data Availability Statement

The raw data supporting the conclusions of this article will be made available by the authors, without undue reservation.

## Ethics Statement

This study was approved by the Institutional Review Board of McGill University (study number A06-B25-17B). The patients/participants provided their written informed consent to participate in this study. Written informed consent was obtained from the individual(s) for the publication of any potentially identifiable images or data included in this article.

## Author Contributions

RM-O, NI-S, and SB-M collected the experimental data. DF and SB-M analyzed the experimental data, interpreted the findings, and wrote the manuscript. RM-O and NI-S constructively reviewed the manuscript. All authors reviewed and approved the final version of the manuscript.

## Funding

This work was supported by the CIHR operating grant Moving With and Tuning In: A participatory mixed methods study to foster social inclusion of individuals with dementia and their carers (CIHR-SII-150,704) and Alzheimer Society of Canada (Grant #17C).

## Conflict of Interest

The authors declare that the research was conducted in the absence of any commercial or financial relationships that could be construed as a potential conflict of interest.

## Publisher's Note

All claims expressed in this article are solely those of the authors and do not necessarily represent those of their affiliated organizations, or those of the publisher, the editors and the reviewers. Any product that may be evaluated in this article, or claim that may be made by its manufacturer, is not guaranteed or endorsed by the publisher.
